# The impact of Middle Eastern Origin, HIV, HCV, and HIV/HCV co‐infection in the development of hypovitaminosis D in adults

**DOI:** 10.1002/mgg3.475

**Published:** 2018-09-13

**Authors:** Saad Warraich, Aven Sidhu, Michelle Hou, Osamah Alenezi

**Affiliations:** ^1^ Vancouver Virology Centre Vancouver BC Canada; ^2^ CIHR HIV Clinical Trials Network Vancouver BC Canada

**Keywords:** adults, Hepatitis C Virus, Human Immunodeficiency Virus, hypovitaminosis D, Middle East

## Abstract

**Background:**

A relationship between hypovitaminosis D and infection with HIV and HCV has been established in the scientific literature. Studies comparing these illnesses to other risk factors for development of hypovitaminosis D, such as being of Middle Eastern origin, have been lacking. The goals of this study were: (a) to document vitamin D levels in groups of individuals at high risk of developing its deficiency, (b) analyze the data collected to numerically determine which group had the lowest average vitamin D levels, and (c) discuss the impact of the findings and offer possible explanations.

**Methods:**

This retrospective observational study involved reviewing medical charts and documenting recent vitamin D levels. Our subgroups were: (a) individuals infected with HIV, (b) individuals infected with HCV, (c) individuals co‐infected with HIV/HCV, and (d) people of Middle Eastern origin. The gathered data was subsequently subjected to statistical analysis.

**Results:**

People of Middle Eastern origin were found more likely to be vitamin D deficient as compared to those infected with HIV, HCV, or co‐infected with both HIV and HCV.

**Conclusion:**

This suggests that genetic and environmental factors unique to otherwise healthy Middle Eastern people are more detrimental, in terms of developing hypovitaminosis D, than being chronically infected with the aforementioned illnesses.

## 1 INTRODUCTION

Vitamin D is a fat‐soluble vitamin involved mainly in calcium homeostasis in the body. Relatively recently, vitamin D has also been shown to have immunomodulatory effects; its receptors have been found on monocytes, macrophages, as well as B and T lymphocytes (Holick, 2007). Synthesis of vitamin D begins in the skin with the conversion of 7‐dehydrocholestrol to cholecalciferol under the influence of Ultraviolet B radiation. This molecule is hydroxylated to 25‐hydroxycholecalciferol in the liver, which itself consequently gets hydroxylated, mostly in the kidneys, to 1,25‐dihydroxycholecalciferol. In addition to this, vitamin D is also acquired through diet.

The Endocrine Society (ENDO) and the International Osteoporosis Foundation (IOF) recommends that levels of 25(OH)D be at or above 75 nmol/L (Dawson‐Hughes et al., 2010; Holick et al., 2011). A low level of vitamin D is implicated in many disease processes (Allavena et al., 2012). It has been shown that osteomalacia and osteoporosis are more common in individuals with hypovitaminosis D (Dawson‐Hughes et al., 2005). Moreover, low levels are also linked with cardiovascular disease and certain malignancies (Souberbielle et al., 2011).

Its deficiency is widely prevalent in various groups of individuals globally (Palacios & Gonzalez, 2014). Among the subpopulations commonly deficient are those infected with Human Immunodeficiency Virus (HIV), Hepatitis C Virus (HCV), as well as those co‐infected with HIV and HCV (Arteh, Narra, & Nair, 2010; Dao et al., 2011; Mandorfer et al., 2013). Dao et al. (2011) discovered that 70.3% of HIV‐infected individuals were found to have low vitamin D levels in their selected sample of patients. Arteh et al. (2010) found that 95.3% of patients from their hepatitis C cirrhosis cohort had vitamin D deficiency. Whereas, Terrier et al. (2011) found 85% of HIV‐HCV co‐infected patients to be deficient. Vitamin D deficiency has shown to play a key role in the course of these illnesses (Lange et al., 2011; Mandorfer et al., 2013). For instance, low vitamin D levels have been associated with poor response to therapy with pegylated interferon‐alfa plus ribavirin in patients infected with HCV genotype 2 and 3; 50% versus 81% sustained virologic response was observed for patients with and without severe vitamin D deficiency, respectively (Lange et al., 2011). Another well‐known group of people that are found to be deficient are individuals of Middle Eastern origin (van Schoor & Lips, 2011). In a study, vitamin D levels in Saudi Arabian students and older people was found on average to be around 10 nmol/L (Sedrani, Elidrissy, & El Arabi, 1983).

In general, deficiency can be attributed to certain factors such as lifestyle choices, diet, some disease states, and intake of certain medications. Genetic causes of developing hypovitaminosis D have also been identified. In their study involving exclusively Caucasian individuals, Wang et al. (2010) found that variants near genes concerned with cholesterol synthesis, hydroxylation, and vitamin D transport lead to a significantly increased risk of developing vitamin D insufficiency.

The aim of this study was to rank four factors that are known to be implicated in the development of hypovitaminosis D. Namely, these four factors are: (a) HIV infection, (b) HCV infection, (c) HIV‐HCV co‐infection, and (d) Middle Eastern origin. By setting out to accomplish this task, the hope was to highlight the relative contribution of each of these factors in developing vitamin D deficiency and thereby facilitate discourse about the course of action moving forward. Knowing which factor is most dominant will enable discussion on policy change considerations where applicable, such as those related to vitamin D screening, and also it will spark enthusiasm for research exploring that factor further.

## 2 METHODS

This retrospective observational study looked at vitamin D levels in people attending an infectious diseases clinic in Vancouver, BC. The medical charts of 266 patients were anonymized and reviewed to tabulate vitamin D levels. Individuals were divided into the following four categories: (a) infected with HIV, (b) infected with HCV, (c) co‐infected with HIV and HCV, and (d) individuals of Middle Eastern background without HIV or HCV. The respective number of people in each category was as follows: (a) 26, (b) 46, (c) 38, and (d) 156.

When determining vitamin D levels, the bone marker 25‐hydroxyvitamin D was used. The total amount of 25‐hydroxyvitamin D is represented by the sum of 25‐hydroxylated vitamin D2 and vitamin D3 species. The values indicating low, normal, high, and toxic were determined by local lab services. Consequently, we defined vitamin D levels <75 nmol/L as low, 75–149 nmol/L as normal, 150–200 nmol/L as high, and above 200 nmol/L as toxic. The collected data was then subjected to statistical analysis; the software used for this purpose was Microsoft Excel version 14.0.7015.1000. The average vitamin D level was calculated for people in each category; the corresponding confidence interval and p value was also computed. The resultant values for the HIV, HCV, and co‐infected subgroups were then compared against the Middle Eastern subgroup.

## 3 RESULTS

**Figure 1:**
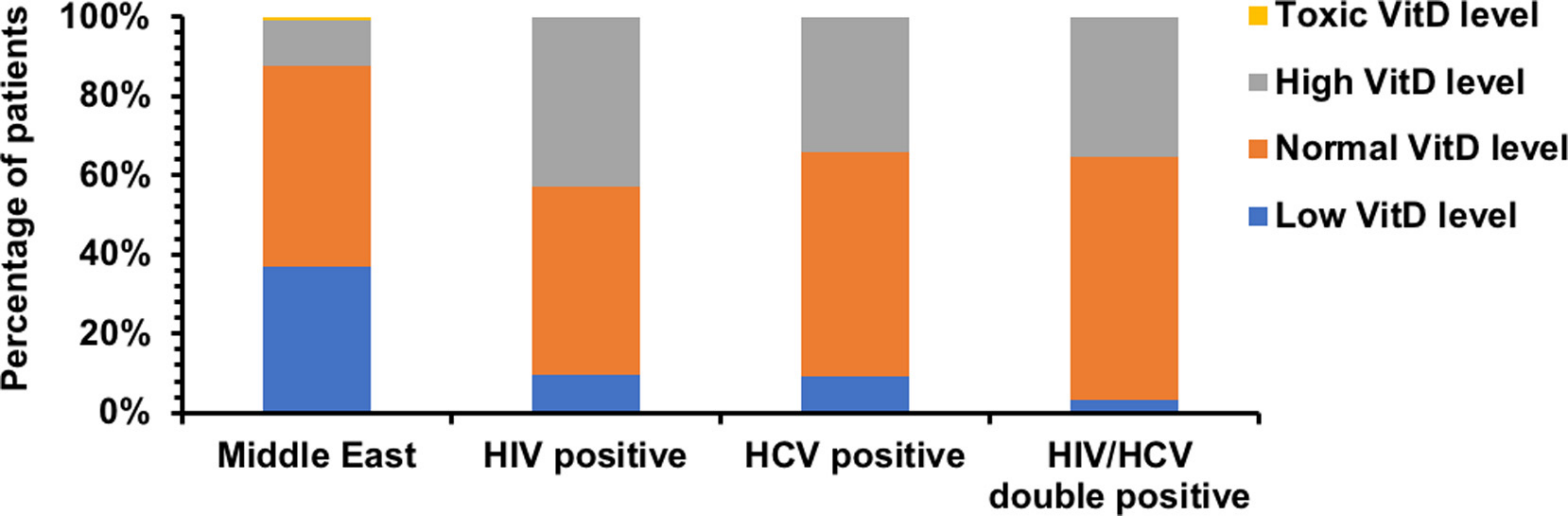
Proportion of individuals with low, normal, high, or toxic vitamin D levels in each of the subgroups

Our results showed that people of Middle Eastern background had, on average, lower levels of vitamin D than those infected with HIV, HCV, or those co‐infected with both HIV and HCV. It was found that 7.7% of HIV, 6.5% of HCV, and 2.6% of co‐infected individuals were vitamin D deficient, as compared to 35.3% of Middle Eastern individuals. The mean value of vitamin D in each of our subgroups was as follows: 64.81 nmol/L (*n* = 26; CI = 54.58–75.03; *p *= <0.05) in the HIV subgroup, 72.83 nmol/L (*n* = 46; CI = 59.67–85.98; *p* < 0.05) in the HCV subgroup, 65.18 nmol/L (*n* = 38; CI = 54.08–76.29; *p* < 0.05) in the co‐infected subgroup, and 43.02 nmol/L (*n* = 156; CI = 38.08–47.96) in the Middle Eastern subgroup. These findings are presented in Table 1 and depicted graphically in Figure 1. Females in the HIV, co‐infected, and Middle Eastern subcategories had on average lower vitamin D levels than their male counterparts (see Table 2). There were only three individuals with toxic levels of vitamin D; one person was from the Middle Eastern subgroup, and the other two belonged to the HCV subgroup. There were no females with hypovitaminosis D in HIV and HCV subcategories.

**Table 1:** Vitamin D statistics for HIV, HCV, co‐infected, and Middle Eastern subgroups

Subgroups	HIV	HCV	Co‐infected	Middle Eastern
Proportion found to be deficient (%)	7.7	6.5	2.6	35.3
Mean Vitamin D (nmol/L)	64.81	72.83	65.18	43.02
Sample size	26	46	38	156
95% confidence Interval (nmol/L)	54.58–75.03	59.67–85.98	54.08–76.29	38.08–47.96

**Table 2:** Distribution of average vitamin D levels according to gender among the sample populations

HIV (nmol/L)		HCV (nmol/L)		Co‐infected (nmol/L)		Middle Eastern (nmol/L)	
Males	Females	Males	Females	Males	Females	Males	Females
68.14	50.80	65.94	88.57	69.39	46.57	41.63	33.99

## 4 DISCUSSION

Hypovitaminosis D among individuals afflicted with HIV is a widely discussed topic in the scientific literature (Adeyemi et al., 2011; Allavena et al., 2012; Dao et al., 2011; Mehta et al., 2010; Mueller et al., 2010; Vescini et al., 2011; Viard et al., 2011). The use of certain antiretroviral drugs has been implicated in developing hypovitaminosis D in this population; namely, ritonavir‐ and efavirenz‐containing regimens have been linked with low levels of vitamin D (Dao et al., 2011). It is worth mentioning that vitamin D deficiency has been independently linked to all‐cause mortality and AIDS‐defining events in HIV‐infected individuals (Viard et al., 2011). Chronic hepatitis C infection, particularly which progresses to liver cirrhosis, is also associated with the development of vitamin D deficiency (Arteh et al., 2010). As mentioned earlier, vitamin D deficiency leads to poor response rates to treatment in those infected with hepatitis C (Lange et al., 2011). Interestingly, another group of individuals that are stricken by hypovitaminosis D are otherwise healthy Middle Eastern people (van Schoor & Lips, 2011). One of the more prevalent problems faced by persons from the Middle East due to vitamin D deficiency, and subsequent osteomalacia, is chronic low back pain (Al Faraj & Al Mutairi, 2003).

Our study showed that a greater percentage of healthy Middle Eastern people have vitamin D deficiency as compared to persons infected with HIV, HCV, and those co‐infected with these two illnesses. Also, we discovered that the mean vitamin D levels are lower in this group than the other three. These results suggest that factors unique to healthy Middle Eastern people are more influential, in the development of vitamin D deficiency, than chronic infection with HIV, HCV or co‐infection with HIV/HCV. We believe possible reasons for this increased vulnerability can broadly be attributed to environmental and genetic causes specific to the Middle Eastern group.

Environmental factors could include daytime outdoor exposure, clothing, and diet. It is well known that people in the Middle Eastern region avoid daytime outdoor exposure due to the intense heat. Clothing that covers most of the body is also used to protect against the sunlight. Moreover, culture and religion also dictate clothing choices; for instance, many women in Middle East can be seen wearing hijab or niqab (face veil). It was found that the vitamin D level of Turkish women with Western‐style clothing was on average 56 nmol/L; for those who wore hijab it was 32 nmol/L, whereas for those who wore the niqab, it was only 9 nmol/L (Alagöl et al., 2000; Atli, Gullu, Uysal, & Erdogan, 2005). Similar pattern was found in Jordan with the values being 37nmol/L, 28nmol/L, and 24 nmol/L in women who wore Western‐style clothing, the hijab, and the niqab, respectively (Mishal, 2001). Additionally, dietary precaution in order to offset these influences is usually not taken. In Qatar, food is not currently fortified with vitamin D (Hamilton, Grantham, Racinais, & Chalabi, 2009). Having mentioned this, data regarding this matter for other countries within the Middle East is presently sparse.

Genetic factors leading to low levels of vitamin D in the Middle Eastern population could be presumed to include individual or combinations of genes involved in synthesis, reabsorption, or utilization of vitamin D or its precursors. Although studies exploring genetic causes of vitamin D deficiency in Middle Eastern people have not been conducted, studies involving individuals of Caucasian descent are available. In one study, it was found that variations in genes involved in cholesterol synthesis, hydroxylation, and vitamin D transport predispose to vitamin D insufficiency (Wang et al., 2010). It is on the basis of such findings that we postulate that similar genetic mechanisms might be at play in Middle Easterners as well.

In conclusion, the findings and the associated discussion presented by this preliminary work yearns further research focused on quantifying and ranking factors unique to Middle Eastern individuals that lead to hypovitaminosis D. Due to the important association of vitamin D with bone health, immunomodulation, cardiovascular disease, and malignancy, it is hoped that our study shifted some attention to the Middle Eastern population—a privilege traditionally apportioned to other high‐risk groups such as those infected with HIV and HCV.

## ETHICAL STATEMENT

This project was exempt from Research Ethics Board (REB) review as per Tri‐Council Policy Statement 2: Ethical Conduct for Research involving Humans (TCPS 2) 2014, Article 2.4.

## CONFLICT OF INTEREST

The authors declare that there is no conflict of interest regarding the publication of this paper.
